# Epidemiology of total hip and knee replacement: a cross-sectional study

**DOI:** 10.1590/S1679-45082013000200011

**Published:** 2013

**Authors:** Mario Lenza, Silvia de Barros Ferraz, Dan Carai Maia Viola, Reynaldo Jesus Garcia, Miguel Cendoroglo, Mario Ferretti

**Affiliations:** 1 Hospital Israelita Albert Einstein, São Paulo, SP, Brazil

**Keywords:** Arthroplasty, replacement, knee/epidemiology, Arthroplasty, replacement, knee/adverse effects, Arthroplasty, replacement, hip/ epidemiology, Arthroplasty, replacement, hip/adverse effects, Risk factors, Cross-sectional studies

## Abstract

**Objective:**

To describe the epidemiologic characteristics and adverse events of patients submitted to total hip and total knee replacement.

**Methods:**

A cross-sectional study retrospectively assessing medical chart data of all total hip and total knee replacements performed at a private hospital, between January 2007 and December 2010 Patients submitted to total hip and total knee replacement, with consent of surgeons were included. Incomplete records and/or missing data of the hospital database were excluded. The categorical variables analyzed were age, gender, type of arthroplasty (primary or secondary), type of procedure, duration of surgery, use of drains, risk of infection, compliance to protocol for prevention of deep venous thrombosis and embolism pulmonary, and compliance to the protocol for prevention of infection. The outcomes assessed were adverse events after surgery.

**Results:**

A total of 510 patients were included; in that, 166 admissions for knee replacements (92 male) and 344 admissions for hip replacements (176 female). The mean age of patients was 71 years (range 31-99 years). Adverse events were reported in 76 patients (14.9%); there was no correlation between assessed variables and number of complications.

**Conclusion:**

The results showed no individual factors favoring complications in patients submitted to total hip and total knee replacement; hence, surgeons should consider prophylaxis to avoid complications.

## RESUMO

**Objetivo:**

Descrever as características epidemiológicas e os eventos adversos dos pacientes submetidos aos procedimentos de artroplastia de quadril e joelho.

**Métodos:**

Estudo transversal, com avaliação retrospectiva de prontuários médicos de todas artroplastias de quadril e de joelho realizadas entre janeiro de 2007 e dezembro de 2010, em hospital privado. Foram incluídos pacientes adultos submetidos à artroplastia total do quadril ou do joelho, com anuência dos cirurgiões. Foram excluídos os pacientes com prontuários incompletos ou perdidos na base de dados do hospital. As variáveis categóricas analisadas foram: idade, gênero, tipo de artroplastia (primária ou secundária), tipo de procedimento, tempo de cirurgia, uso de drenos, risco de infecção, adesão ao protocolo para prevenção de trombose venosa profunda e embolia pulmonar, e adesão ao protocolo para prevenção de infecção. Os desfechos avaliados dos pacientes foram eventos adversos ocorridos após a cirurgia.

**Resultados:**

Foram incluídos 510 pacientes, sendo 166 hospitalizações para artroplastia do joelho (92 do gênero masculino) e 344 hospitalizações para artroplastia do quadril (176 do gênero feminino). A média da idade dos pacientes foi 71 anos (variação 31 a 99 anos). Os eventos adversos ocorreram em 76 pacientes (14,9%); não foi encontrada correlação entre as variáveis avaliadas e o número de complicações.

**Conclusão:**

Os dados não demonstraram fatores individuais que favorecessem complicações de pacientes submetidos à artroplastia do quadril e joelho; portanto, os cirurgiões têm que considerar administrações profiláticas para evitar complicações.

## INTRODUCTION

Total hip replacement (THR) and total knee replacement (TKR) are indicated for treating chronic refractory joint pain and some types of proximal femoral fractures. The most frequent condition for both THR and TKR is osteoarthritis (OA); other diseases that commonly lead to THR and TKR are rheumatoid arthritis, fractures and avascular necrosis^(1,2)^. The data of Brazilian patients undergoing THR or TKR showed that osteoarthritis was the primary indication for both procedures and hypertension was the prevalent comorbity among patients^(3)^.

Joint replacement is a reasonably safe intervention that can result in considerable pain relief and reduce disability for allowing the new joint to work normally^(4–7)^.

The use of these procedures has increased due to the good results obtained. Taking into consideration only the demographic changes, it is estimated that the number of indications for THR will increase by 40% by 2021 in the United Kingdom^(8)^.

A cross-sectional study taking into account data from 2006 to 2007 reported there were 62,196 hospitalizations for THR and TKR in Canada, with an overall incidence of 81.2 per 100,000 people per year^(9)^.

A literature review evaluated the prevalence and incidence rates for THR and TKR; the authors found the rates of indication and utilization of both interventions vary according to socioeconomic status and healthcare systems, patient preferences and prevalence of osteoarthritis. The authors also concluded that future studies should be conducted to examine the causes of these differences^(7)^.

## OBJECTIVE

The objective of this study was to describe the epidemiologic characteristics of total hip replacement and total knee replacement interventions performed at a Brazilian private hospital, according to patient demographics and tendencies in the clinical practice over time.

## METHODS

Institutional review board approval (number 5162) was obtained to retrospectively review medical records and analyze pertinent data. Data of every THR and TKR performed at the *Hospital Israelita Albert Einstein* (HIAE), São Paulo, Brazil, were prospectively collected and stored from January 2007 to December 2010.

The inclusion criteria were adults (18 or more years) who underwent primary or revision THR and TKR at our hospital and had a consent form signed by surgeons. The exclusion criteria were incomplete records and missing data from the hospital database.

### Patient demographics

The following variables were recorded and analyzed: age (stratified into 10-year groups, beginning with 0 to 4 years and ending with >94 years), gender, type of arthroplasty (primary or secondary), type of procedure (THR or TKR), duration of surgery time, use of drains, compliance to the protocol for infection prevention^(10)^ and to the protocol for deep venous thrombosis (DVT) and pulmonary embolism (PE) prevention^(11)^.

### Types of outcomes

The postoperative adverse events of all patients were gathered and recorded using a standardized form. The surgeons and nurses were audited on their gathering rate of data on adverse events and re-oriented on proper completion of data. All patients and their primary care physicians were instructed to contact the hospital in case of any suspicion of infection. Those who did not come for follow-up were contacted by telephone. Infection, DVT, PE, failure of treatment and all-cause mortality were assessed as adverse events.

All cases of potential superficial, deep local infection and systemic infections were recorded and analyzed according to the definitions and guidelines (all versions) used in the United Kingdom^(10)^. We also calculated the surgical site infection (SSI) risk index, a score used to predict a surgical risk of acquiring an infection in the surgical site. The SSI risk index, ranging from 0 to 3, consists of data obtained from three risk factors: a) American Society of Anesthesiologists' physical status classification (ASA); b) wound classification and; c) duration of operation. Patients who underwent surgical interventions with a risk index of 3 had a higher risk of developing SSI than those with a score of 0^(12,13)^.

For patients suspected of symptomatic DVT, bilateral ultrasound Doppler and/or venography were performed according to the standardized technique previously described in the literature^(14,15)^. PE was diagnosed by ventilation-perfusion scintigraphy, pulmonary angiography, or spiral computed tomography.

Treatment failure was considered when patients were submitted to a non-routine secondary surgical intervention for symptomatic aseptic or septic loosening, instability, or dislocation after 30 days of the first surgery.

### Data and statistical analysis

Data of all primary and revision THR and TKR from January 2007 to December 2010 were gathered from the hospital database and analyzed using the statistical package Excel (Microsoft Office Excel 2007.Ink). The prevalence rates were given as percentages. Pearson's χ^2^ test was used to assess discrete data; the significance of various risk factors was calculated by multivariate logistic regression analysis in which the odds ratio and 95% confidence intervals were calculated.

## RESULTS

### Characteristics of the study population

**Figure 1:**
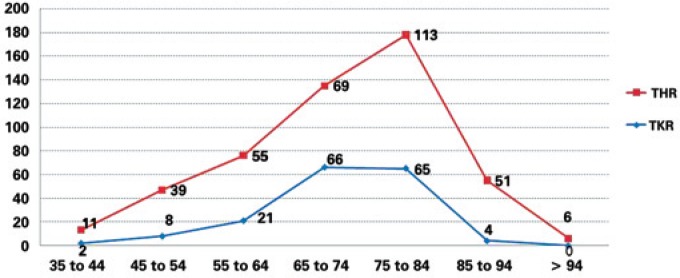
Age (stratified by 10-year age groups, beginning at 35 and ending at >94 years) – 2007 to 2010

The study population consisted of 510 patients; there were 166 hospitalizations for knee replacements (92 male) and 344 admissions for hip replacements (176 female). The mean age of patients undergoing TKR was 71 years (range 31 to 87 years) and also 71 years (range 33 to 99 years) for THR. Table 1 describes the demographic characteristics of patients per year. The age distribution of hip and knee replacement patients was similar, with the majority of patients being 65 years or older (81.3% for TKR and 69.5% for THR). Only a small proportion of patients for both procedures (TKR and THR) were aged less than 45 years (1.2% for TKR and 3.2% for THR) (Figure 1).

**Table 1:** Demographic data

Patients		Knee replacement		Hip replacement	
		Jan 2007 to Dec 2008	Jan 2009 to Dec 2010	Jan 2007 to Dec 2008	Jan 2009 to Dec 2010
Gender					
	Male n (%)	18 (31.0)	74 (68.5)	46 (37.1)	122 (55.5)
	Female n (%)	40 (69.0)	34 (31.5)	78 (62.9)	98 (44.5)
Age					
	Mean (SD)	71 (9.7)	72 (9.2)	73 (13.5)	72 (13.8)
	Range	50-87	31-87	38-97	33-99
Primary diagnosis		**n (%)**	**n (%)**	**n (%)**	**n (%)**
	Osteoarthritis	46 (79.3)	95 (88.0)	52 (41.9)	117 (53.2)
	Fracture	0 (0)	0 (0)	54 (43.5)	78 (35.5)
	Osteonecrosis	1 (1.7)	1 (0.9)	6 (4.8)	10 (4.5)
	Rheumatoid arthritis	1 (1.7)	1 (0.9)	4 (3.2)	9 (4.1)
	Others	10 (17.2)	9 (8.3)	8 (6.5)	6 (2.7)
Type of joint replacement					
	Primary	46 (79.3)	98 (90.7)	115 (92.7)	203 (92.3)
	Revision	12 (20.7)	10 (9.3)	9 (7.3)	17 (7.7)
Clinical condition					
	Hypertension	36 (62.1)	64 (59.3)	64 (51.6)	93 (42.3)
	Congestive heart failure	13 (22.4)	19 (17.6)	29 (23.4)	34 (15.5)
	Diabetes	11 (19.0)	24 (22.2)	17 (13.7)	45 (20.5)
	Cerebrovascular accident	0 (0)	2 (1.9)	7 (5.6)	10 (4.5)
	Benign or malignant tumors	1 (1.7%)	7 (6.5%)	14 (11.3)	15 (6.8)
	Total of patients	58	108	124	220

SD: standard deviation.

The main preoperative diagnosis for patients was osteoarthritis (84.9% for TKR and 49.1% for THR). Moreover, most admissions for joint replacement in our population were for primary procedures (86.7% for TKR and 92.4% for THR). The most frequent preoperative medical condition diagnosed in all patients was hypertension (50.4%) (Table 1).

All patients received deep venous thrombosis prophylaxis and prophylactic antibiotics according to the current literature^(10,11)^.

Table 2 shows some patient characteristics and interventions. Most TKR patients were seen in intensive care unit (51.2%) and most THR patients were admitted to the Orthopedics Unit (51.2%). The mean length of hospital stay was 8 days (range 2-40 days) for TKR patients and 10 days (range 2-230 days) for THR patients. The commonest type of anesthesia was a combination of spinal anesthesia and sedation, given to 276 (54.1%) patients. Closed suction drains were used in 458 (89.8%) patients. Most patients were classified as grade 1 (50.8%) in terms of SSI risk index.

**Table 2:** Characteristics of patients and interventions

Characteristics		Knee replacement		Hip replacement	
		Jan 2007 to Dec 2008	Jan 2009 to Dec 2010	Jan 2007 to Dec 2008	Jan 2009 to Dec 2010
Postoperative care		**n (%)**	**n (%)**	**n (%)**	**n (%)**
	Intensive care unit	31 (53.5)	54 (50.0)	88 (71.0)	66 (30.0)
	Stepdown unit	1 (1.7)	12 (11.1)	3 (2.4)	11 (5.0)
	Orthopedic unit	26 (44.8)	42 (38.9)	33 (26.6)	143 (65.0)
Length of hospital stay (days)					
	Mean (SD)	8 (11.7)	7 (15.9)	8 (5.6)	8 (4.1)
	Range	2-40	2-29	2-106	2-220
Type of anesthesia		**n (%)**	**n (%)**	**n (%)**	**n (%)**
	General	11 (19.0)	22 (20.4)	46 (37.1)	87 (39.5)
	Spinal	2 (3.4)	3 (2.8)	0 (0)	3 (1.4)
	General + spinal	0 (0)	4 (3.6)	19 (15.3)	28 (12.7)
	Sedation + spinal	42 (72.4)	76 (70.4)	56 (45.2)	102 (46.4)
	Not reported	3 (5.2)	3 (2.8)	3 (2.4)	0 (0)
Drained wound					
	Number of patients	49 (84.5)	104 (96.3)	116 (93.5)	189 (85.9)
SSI risk index					
	Grade 0	16 (27.6)	36 (33.3)	43 (34.7)	91 (41.4)
	Grade 1	32 (55.2)	56 (51.8)	65 (52.4)	106 (48.2)
	Grade 2	5 (8.6)	10 (9.3)	9 (7.3)	23 (10.4)
	Not reported	5 (8.6)	6 (5.6)	7 (5.6)	0 (0)
ASA classification					
	I	6 (10.3)	12 (11.1)	8 (6.5)	30 (13.6)
	II	41 (70.7)	83 (76.9)	84 (67.7)	144 (65.4)
	III	7 (12.1)	7 (6.5)	30 (24.2)	44 (20.0)
	IV	0 (0)	0 (0)	0 (0)	2 (1.0)
	Not reported	4 (6.9)	6 (5.5)	2 (1.6)	0 (0)
Wound classification					
	Not contaminated	57 (98.3)	106 (98.0)	124 (100)	218 (99.1)
	Contaminated or dirty	0 (0)	1 (1.0)	0 (0)	2 (0.9)
	Not reported	1 (1.7)	1 (1.0)	0 (0)	0 (0)
Duration of surgery (hours: minutes)					
	Mean (SD)	2:37	2:25	2:04	2:16
	Range	00:50 to 05:30	01:00 to 05:00	00:35 to 04:40	00:50 to 05:15

Source: *Hospital Israelita Albert Einstein - HIAE.*

SD: standard deviation; SSI: surgical site of infection.

### Assessed outcomes

Some adverse events, such as infection, DVT, PE, failure of treatment and death are shown in table 3. The primary outcome death (all-causes) occurred in four patients of the THR group; DVT or PE were diagnosed in six patients (four in THR and two in TKR group); no patient had failure of treatment.

**Table 3:** Postoperative medical complications

Complication		Knee replacement		Hip replacement	
		Jan 2007 to Dec 2008	Jan 2009 to Dec 2010	Jan 2007 to Dec 2008	Jan 2009 to Dec 2010
All-cause mortality		0	0	0	4
DVT or PE		0	2	1	3
Superficial wound infection		1	1	3	2
Genitourinary					
	Urinary tract infection	2	0	7	0
	Urinary retention	0	0	1	1
	Scrotal swelling	0	0	0	1
Cardiovascular					
	Myocardial infarction	0	0	0	2
	Atrial fibrillation	1	0	1	0
Pulmonary					
	Pneumonia	0	2	0	2
	Respiratory insufficiency	0	0	2	3
	Dyspnea	2	0	0	2
Neurological					
	Confusion	0	1	1	2
Gastrointestinal					
	Stomatitis	0	0	0	1
	Fecaloma	1	0	0	0
	Anemia	1	1	2	3
	Fever with no other symptoms	0	1	0	3
	Other complications	2	1	4	6

DVT: deep venous thrombosis; PE: pulmonary embolism.

In our cohort of patients, seven (five in the THR and two in the TKR group) presented superficial wound infection, which demanded a four-week antibiotic therapy, but there was no need to change treatment or new surgical procedure. Fifty-nine patients (11.6%) experienced other complications; genitourinary conditions were the most prevalent complications presented by patients (Table 3).

The multivariate logistic regression analysis for patients undergoing THR revealed that ISS (χ^2^=3,962), age (χ^2^=0.191) and comorbidities (χ^2^=0.553) were not statistically significant predictors of complications (p=0.138, p=0.662 and p=0.457, respectively).

The data available for patients submitted to TKR were insufficient; therefore no multivariate logistic regression analysis was performed. Furthermore, there were no individual significant predictors of complications.

## DISCUSSION

In this study information was gathered from many surgeons from the database on hip and knee replacement to highlight trends and different techniques for the procedures. The results show demographic characteristics similar to those of published studies^(9,16–18)^; that is, the majority of patients were aged over 65 years, osteoarthritis was the most common indication for surgery and the most frequent clinical comorbidity was hypertension. Regarding gender, the results differ from the literature - the majority of our patients were male; however, when sex distribution was divided by procedure, there were more women for THR.

Our outcomes support some recommendations regarding total joint replacement^(19–22)^. Although THR and TKR are relatively safe interventions, 76 medical complications (related or not to the surgical procedure) were observed, demonstrating that surgeons should be cautious.

The mortality rate was very low - four patients described as follows: the first patient (initials IPB, female, 87 years old) had metastatic adenocarcinoma, required THR for femoral neck fracture and died 46 days after surgery. The second patient (initials MAXB, female, 89 years old) had femoral neck fracture and no co-morbid conditions, and passed away 3 days after surgery; the third (initials EC, male, 84 years old) was a chronic patient with respiratory insufficiency (chronic obstructive pulmonary disease - COPD) and acute renal failure, who underwent THR for femoral neck fracture and died during the surgery. The fourth patient (initials APLS, male, 99 years old) presented atrial fibrillation, had femoral neck fracture, and died 19 days after surgery.

In our view, it is important to be aware of the data from this cohort study, such as complications, infections, costs and admission. Some useful data were described in this study, and based on our results some strategies could be established to reduce complication rates.

When reporting on some characteristics of patients submitted to THR and TKR at a private hospital, it is essential to compare with other hospitals and countries. This analysis is complex because of diversity of population among countries, reporting methods, study design, periods of studies, different healthcare systems. Nevertheless, cross-sectional and qualitative analyses are fundamental to improve care in health systems.

The increased health-related costs all over the world and the restrictions on economic resources led to great emphasis on cost-effective procedures in health^(23–25)^. The orthopedic implants, mostly knee and hip prostheses, account for more hospital expenses than any other procedures. Despite elevated hospital costs and more orthopedic implants, decrease in infection and complication rates, healing of the orthopedic diseases and better patient satisfaction have not been observed.

Aiming to enhance quality of clinical practice, to support decision-making processes, to offer appropriate usage of medical technologies, to establish a better and more productive management, in addition to standardize treatments and avoid waste of resources, protocols were implemented at our hospital. One of the most important characteristics of these protocols is to provide an overview of each procedure to be performed, with its expected results, so that they can be monitored by the clinical team and by the patients. Future investigations will be conducted to assess effectiveness of these protocols.

In order to reduce the costs and better use the hospitals resources, it is essential to have a multidisciplinary team. Professionals with diverse backgrounds, including those from the financing and human resources departments, nurses and doctors, amongst others, should share information about their specific areas and continuously assess their routines, and make changes, whenever necessary. By discussing hospital procedures and their specific daily activities, as well as using evidence-based studies in their meetings to support decision-making, the teams will be able to better use the resources and to avoid unnecessary expenses.

Because of our study design and the small number of patients undergoing THR and TKR at the hospital, no significant conclusions could be drawn on this group. In addition, there are several limitations to the results obtained. First, the data were analyzed retrospectively from our database, which means there are some concerns about data collection. Second, the cross-sectional study is descriptive and typically there is no hypothesis, hindering causal inferences. Moreover there are many aspects that influence the incidence, prevalence and complications of THR and TKR for OA. We are not able to presume if the characteristics described in this study will maintain the same rates. Finally, there are some reservations about the findings since the study was limited and did not gather data from other private or public hospitals.

## CONCLUSION

Our results found no individual significant predictors of complications in patients submitted to THR and TKR. When taking into account joint replacement, our data showed that orthopedic surgeons and patients should be aware of some adverse events that could occur. Hence surgeons must consider prophylactic management to avoid some complications.
